# Impact of pulsed stimulation on objective and subjective visual acuity measurements in nystagmus

**DOI:** 10.1007/s10633-025-10060-z

**Published:** 2025-11-04

**Authors:** Elisabeth V. Quanz, Khaldoon O. Al-Nosairy, Francie H. Stolle, Juliane Kuske, Sven P. Heinrich, Michael Bach, Michael B. Hoffmann

**Affiliations:** 1 Ophthalmic Department, Otto-Von-Guericke University Magdeburg, Leipziger Str. 44, 39120 Magdeburg, Germany; 2 Eye Center, Medical Center – University of Freiburg, Freiburg, Germany; 3 Faculty of Medicine, University of Freiburg, Freiburg, Germany; 4 Center for Behavioral Brain Sciences Magdeburg, Magdeburg, Germany

**Keywords:** VEP, Nystagmus, Visual acuity, Fixation instability

## Abstract

**Purpose:**

Quanz et al. (Sci Rep 14:16797, 2024) reported that participants with nystagmus had higher objective visual evoked potential visual acuity estimates (VA_VEP_) by 0.12 logMAR relative compared to standard psychophysical VA (VA_Psych_Stat_). The cause of this modest, but significant VA_VEP_ overestimation remains unclear. Here we investigated its association with the pattern-pulse stimulation mode applied for steady state VEP recording for VA_VEP_ estimation. Specifically, we tested whether psychophysical visual acuity to pulsed optotypes (VA_Psych_Pulsed_) also exceeds standard optotype VA_Psych_Stat_.

**Methods:**

Twelve participants with nystagmus were included in this analysis. VA_VEP_ was determined for pattern-pulse steady-state VEP stimulation (Quanz et al. in Sci Rep 14:16797, 2024) using EP2000, psychophysical VA was determined to stationary (VA_Psych_Stat_) and to pulsed (VA_Psych_Pulsed_) Landolt-C optotypes employing a modified version of the Freiburg Vision Test (FrACT). Pulsed stimulus timing was identical for VEP and VA (40 ms on and 93 ms off, i.e. at 7.5 Hz). In a separate measurement, fixation stability within the central 4° was determined using microperimetry (Nidek MP‐1), and the eye with the stronger fixation instability was selected for the analysis (12 eyes). LogMAR differences were assessed with a paired t-test and the correlation of fixation stability and VA differences (ΔVA_Psych_ = VA_Psych_Pulsed_ – VA_Psych_Stat_) was tested.

**Results:**

VA_Psych_Stat_ (0.43 ± 0.06 logMAR) and VA_Psych_Pulsed_ (0.45 ± 0.06 logMAR, P = 0.15) did not differ from each other, but from VA_VEP_ (0.26 ± 0.08 logMAR, *P* = 0.02 and *P* = 0.01, respectively). There was no correlation of ΔVA_Psych_ with fixation instability (r^2^ = 0.002, *P* = 0.89).

**Conclusion:**

Pulsed stimulation appears not to be the reason for the VA_VEP_ overestimation in nystagmus. Further research should address whether differences in the spatial stimulus properties might be of relevance, as VA_Psych_ is tested with optotypes, VA_VEP_ with extended patterns.

## Introduction

Objective estimation of visual acuity (VA) is of paramount importance in ophthalmology particularly when standard psychophysical VA (VA_Psych_) might be unfeasible or unreliable. Visual evoked potential based-assessment of visual acuity (VA_VEP_) is regarded to provide a relevant option to obtain objective VA estimates [1, 2]. Still, VEP-based VA estimation is facing challenges in certain conditions e.g., amblyopia [3, 4]. Recently, Quanz et al. [5] reported a small but significant VA_VEP_ overestimation in nystagmus, by on average 0.12 logMAR compared to VA_Psych_, that depended on the degree of fixation instability. In that study VA_VEP_ was estimated with pattern-pulse stimulation, VA_Psych_ with static optotypes. In fact, VEP responses to pulsed stimuli are less reduced by nystagmus-like stimulus movements than those to non-pulsed (pattern-reversal) stimuli [6]. This prompts the question whether the VA_VEP_ overestimation in nystagmus is due to the pulsed stimulation mode. For psychophysical grating acuity, pulsed and non-pulsed conditions have been compared and no difference has been found [7]. Critically, for optotype acuity, this has not yet been investigated. We aimed to fill this gap and tested the hypothesis whether pulsed optotypes yield better VA-values (VA_Psych_Pulsed_) than static (VA_Psych_Stat_), i.e., that the stimulation mode accounts for the mismatch of VA_VEP_ and the routinely acquired VA_Psych_Stat_.

## Methods

This study included participants from Quanz et al. study [5], where a detailed record on the procedures is given. It followed the guidelines of the Declaration of Helsinki, and was approved by the Ethics Committee of the Faculty of Medicine, Otto-von-Guericke University, Magdeburg (153/18). All participants gave written informed consent.

### Participants

For this study, we included data from 12 participants (mean and range of age: 39, 20 − 63y; only the individual’s eye with stronger fixation instability was included) with nystagmus from the Quanz et al. cohort [5], for whom also VA_Psych_Pulsed_ data were available, that had previously not entered analysis and publication. For patient characteristics see Table 1. Causes of nystagmus were idiopathic infantile nystagmus syndrome (INS; n = 5), albinism (AL; n = 5), achiasma (ACH; n = 1) or acquired nystagmus (AN; n = 1). Epilepsy, dizziness, and any eye diseases affecting visual function, e.g., diabetic retinopathy, were exclusion criteria.

**Table 1:** Participant characteristics

ID	Group	Sex	Age [years]	Nystagmus Type	Stereopsis*	BCVA [logMAR]	Eye†	Fixation ± 2° [%]
1	INS	f	21	J/H	No	0.54	OD	42
2	INS	f	42	J/H	No	0.41	OD	86
3	INS	m	29	P/H	No	0.32	OS	37
4	INS	f	56	J/H	No	0.32	OS	99
5	INS	m	51	J/H	Yes	0.28	OS	74
6	AN	f	42	J/H	Yes	-0.07	OD	99
7	AL	f	23	J/H	No	0.47	OD	74
8	AL	m	51	J/H	No	0.73	OS	91
9	AL	m	63	J/H	No	0.69	OS	99
10	AL	f	20	J/H	No	0.61	OS	36
11	ACH	m	22	J/H	No	0.27	OS	88
12	AL	m	40	J/H	No	0.57	OD	43

INS, idiopathic infantile syndrome (excluding albinism and achiasma); AN, acquired nystagmus (cause: hydrocephalus shunt surgery); AL, albinism; ACH, achiasma; f, female; m, male; J, jerk, P, pendular, H, horizontal; *Stereopsis test using Lang test; BCVA [logMAR], best corrected visual acuity in logarithmic minimal angle of resolution; Eye†, eye with stronger fixation instability; OD, right eye; OS, left eye

### VA_VEP_ estimation

Participants wore appropriate refractive correction, procedures followed ISCEV recommendations [1]. The VEP was recorded separately for each eye twice and averaged across repetitions. Freiburg Evoked Potential EP2000 allows for stimulation, recording and analysis of the VEP. Two monitors were used, one viewed by the participant and the other for data monitoring. VEP check-size dependence (size range: 0.048–0.8°) was determined for pattern-pulse steady-state (ss)- VEP stimulation (40 ms on/93 ms off, 7.5 Hz; 50 cd/m^2^ mean luminance and 40% contrast) at a viewing distance of 114 cm as described in Quanz et al. [5] using EP2000 [8]. Signals were 100 k-times amplified and band-pass filtered (0.3, 70.0 Hz low and high cut-off). Subsequently, responses were digitally filtered with a low-pass cutoff of 40 Hz. The VA_VEP_ estimation followed Bach et al. [9]. For each checksize, the spatial frequency was determined (SF [cpd] = 1/√2 × check size). The responses and their significance levels were calculated for each SF. VEP amplitudes and significance-levels at the stimulation frequency were determined with a Fourier-analysis based approach [10–12] and subjected to a heuristic algorithm to determine the spatial frequency limit for VA_VEP_ estimation. The previously published stepwise heuristic algorithm [9] was used to determine the upper SF limit (SF_0_) by extrapolating the log amplitude response to zero [5]. SF_0_ was converted to VEP acuity with an acuity-independent conversion factor [9] [decimal VA_VEP_ = SF_0_/17.6 cpd, which corresponds to logMAR VA_VEP_ = log (SF_0_/17.6 cpd)].

### VA_Psych_ estimation

Both best-corrected VA_Psych_Stat_ and VA_Psych_Pulsed_ were determined twice (18 trials per measurement) for testing at 114 cm using the Freiburg Vision Test (FrACT) with Landolt C optotypes [13]. Pulsed stimulus timing for VA_Psych_Pulsed_ was identical with the VEP pulsed mode (40 ms on / 93 ms off, 7.5 Hz; 102 cd/m^2^ mean luminance and 96% contrast).

### Microperimetry – Fixation stability

The fixation stability within the central 4° was quantified using a fundus-controlled microperimeter (MP-1 microperimeter, Nidek, Padova, Italy). The fundus-motion was tracked at 25 Hz for an epoch of 30 s, where participants were asked to fixate a central target.

### Analysis and statistics

Normality was confirmed with the Shapiro–Wilk test and consequently parametric statistical testing was applied. One-sided paired t-tests were employed to determine whether VA_Psych_Pulsed_ exceeded VA_Psych_Stat_ and VA_VEP_ exceeded VA_Psych_ estimates. Pearson correlation (r^2^) explored how ΔVA_Psych_ (VA_Psych_Pulsed_ – VA_Psych_Stat_) depended on fixation instability within the central 4°.

## Results

**Fig. 1:**
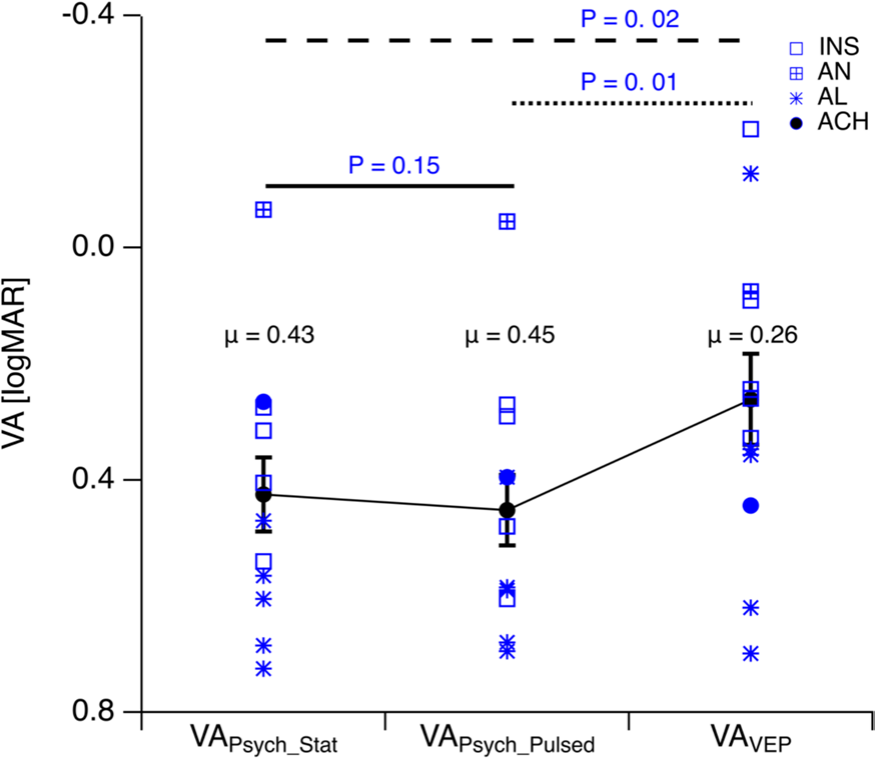
Overview of VAs. (INS: idiopathic infantile syndrome); AN, acquired nystagmus (cause: hydrocephalus shunt surgery); AL, albinism; ACH, achiasma; note inverted y-axis such that better VAs are at top. The average VA (μ) is also shown for each stimulus type

**Fig. 2:**
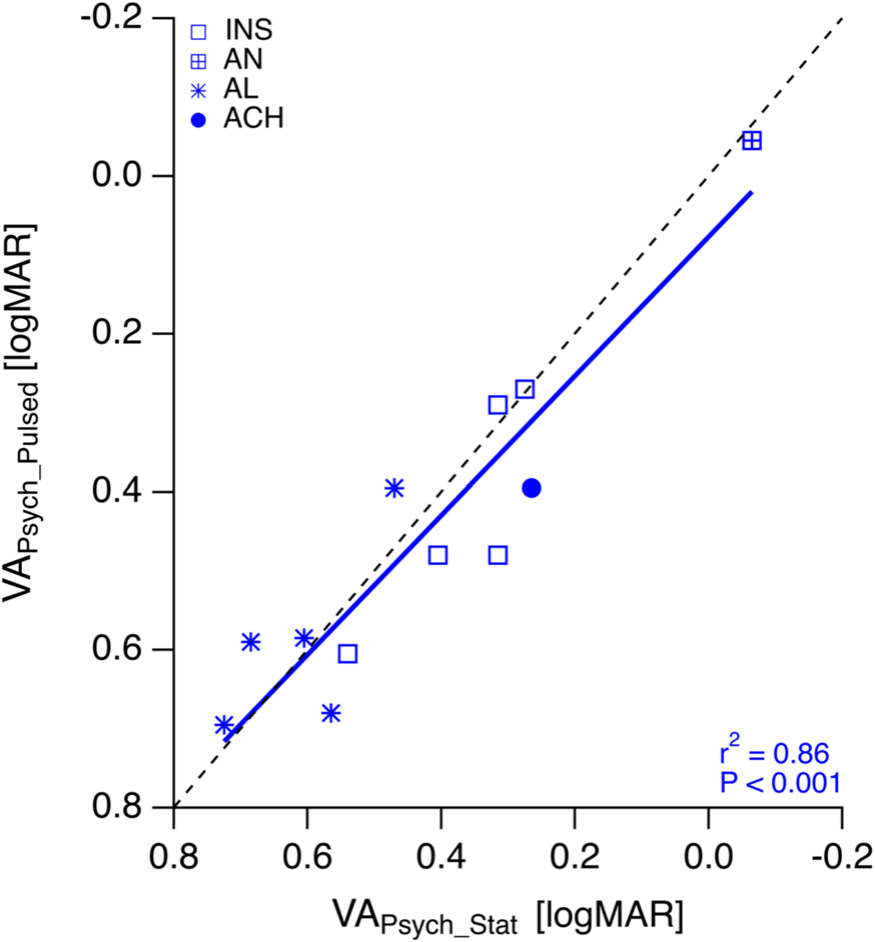
Correlation of VA_Psych_Pulsed_ vs. VA_Psych_Stat_. Both VA measures were highly correlated. For abbreviations see Fig. 1. Dotted line indicates the line of identity; note inverted axes such that better VAs are at top

**Fig. 3:**
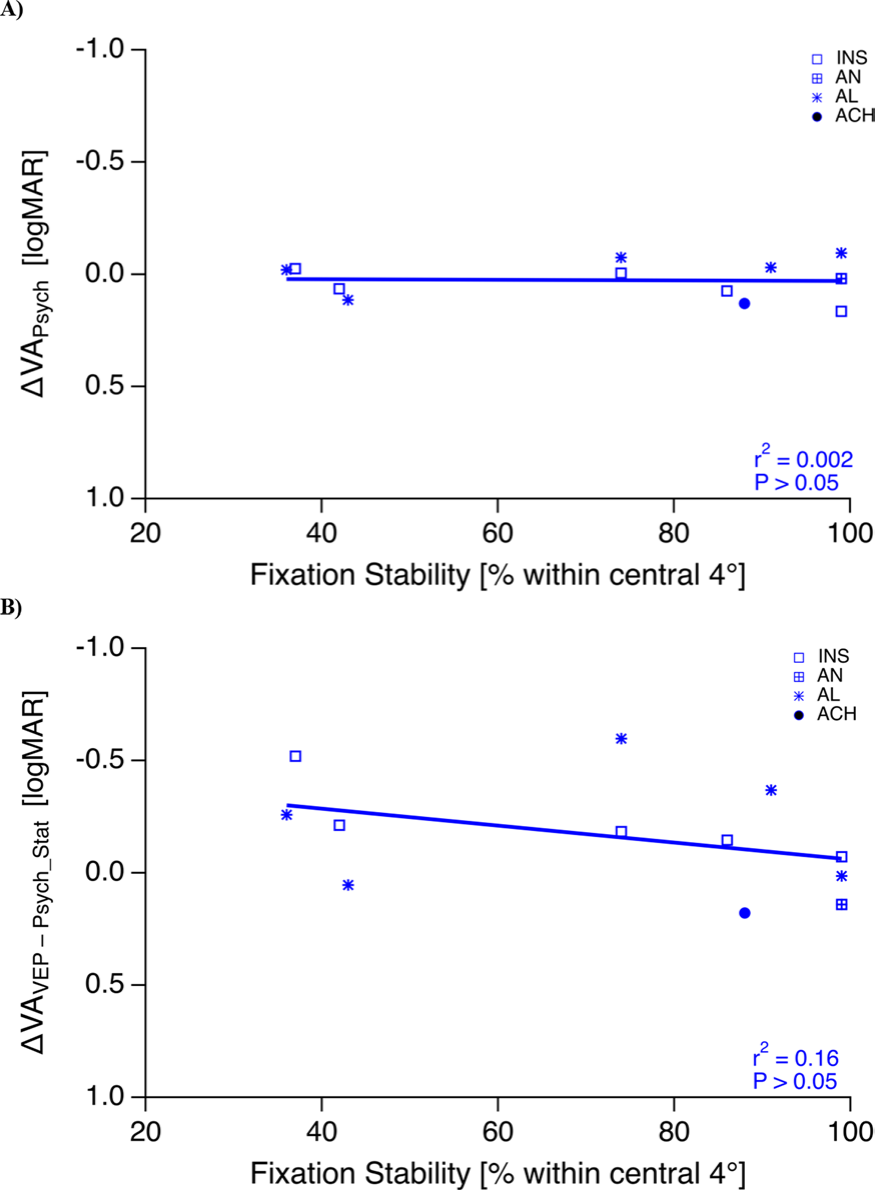
Correlation of fixation stability vs (A) ΔVA_Psych_ = VA_Psych_Pulsed_ − VA_Psych_Stat_ [logMAR] and (B) VA_VEP_ − VA_Psych_Stat_ [logMAR]. No significant correlations were observed. For abbreviations see Fig. 1a

An overview of the mean VAs, VA_Psych_Stat_, VA_Psych_Pulsed_, and VA_VEP_ is given in Fig. 1. We observed a significant overestimation of VA_VEP_ (0.26 ± 0.08 logMAR) compared to both VA_Psych_Stat_ (0.43 ± 0.06 logMAR, *P* = 0.02) and VA_Psych_Pulsed_ (0.45 ± 0.06 logMAR, *P* = 0.01). Notably, VA_Psych_Pulsed_ and VA_Psych_Stat_ did not differ significantly from each other (*P* = 0.15) and were highly correlated with each other (r^2^ = 0.86, *P* < 0.001, Fig. 2). Finally, neither did the difference between VA_Psych_Pulsed_ and VA_Psych_Stat_, nor that between VA_VEP_ and VA_Psych_Stat_ correlate with the degree of fixation instability (ΔVA_Psych_, r^2^ = 0.002, P = 0.89; ΔVA_VEP_—VA_Psych_Stat_, r^2^ = 0.16, *P* = 0.21, Fig. 3 A & B).

## Discussion

We found similar psychophysical visual acuities (VA_Psych_) for static and pulsed optotypes in nystagmus patients, and no dependence on the degree of fixation instability. It is concluded that the previously observed mismatch of VA_VEP_ and VA_Psych_ in nystagmus is not related to the difference in stimulation mode between standard psychophysical VA estimation (‘static’) and VEP based estimation (‘pulsed’).

While we are, to our knowledge, the first to compare psychophysical VA for static vs pulsed optotypes stimulation in nystagmus patients, there is a previous investigation of this issue for psychophysical grating acuity [7]. Dunn et al. report similar findings to the present study; they compared psychophysical static grating VA to tachistoscopically flashed (0.76 ms) patterns and failed to find a difference in nystagmus and healthy controls. Taken together, it appears that pulsed stimulation does not affect psychophysical VA estimates in nystagmus. The lack of comparison to healthy controls and small sample size are a limitation of the present study. Further, the generalizability of the findings across individual subtypes of nystagmus remains limited due to small sizes of subtypes, e.g., albinism. In conclusion, the stimulation time course does not appear to be an explanation of the VA_VEP_ overestimation in nystagmus. In search of an alternative explanation, it should be noted that VEP stimulation employs extended patterns, namely checkerboards, as opposed to optotype stimulation in VA_Psych_ measurements.

Taken together, our report indicates the need for further research into the mechanisms contributing to the slight, but significant VA_VEP_ overestimation in nystagmus given the absence of an effect of pulsed stimulation.

## Data Availability

Data is available upon request to the corresponding author.

## Declarations

### Statement on the welfare of animals

This article does not contain any procedures with animals.
